# Genome-wide analysis of the GLP gene family and overexpression of *GLP1-5–1* to promote lignin accumulation during early somatic embryo development in *Dimocarpus longan*

**DOI:** 10.1186/s12864-023-09201-y

**Published:** 2023-03-21

**Authors:** Zhuoyun Li, Zhuoran Fu, Shuting Zhang, Xueying Zhang, Xiaodong Xue, Yukun Chen, Zihao Zhang, Zhongxiong Lai, Yuling Lin

**Affiliations:** Institute of Horticultural Biotechnology, Fujian Agriculture and Forestry University, Fuzhou, 350002 China

**Keywords:** *D. longan*, *GLP* gene family, Somatic embryogenesis, Expression pattern, Subcellular localization, Lignin

## Abstract

Longan (*Dimocarpus longan* Lour.) is an economically important subtropical fruit tree. Its fruit quality and yield are affected by embryo development. As a plant seed germination marker gene, the germin-like protein (GLP) gene plays an important role in embryo development. However, the mechanism underlying the role of the GLP gene in somatic embryos is still unclear. Therefore, we conducted genome-wide identification of the longan GLP (*DlGLP*) gene and preliminarily verified the function of *DlGLP1-5–1*. Thirty-five genes were identified as longan GLP genes and divided into 8 subfamilies. Based on transcriptome data and qRT‒PCR results, *DlGLP* genes exhibited the highest expression levels in the root, and the expression of most *DlGLPs* was upregulated during the early somatic embryogenesis (SE) in longan and responded to high temperature stress and 2,4-D treatment; eight *DlGLP* genes were upregulated under MeJA treatment, and four of them were downregulated under ABA treatment. Subcellular localization showed that *DlGLP5-8–2* and *DlGLP1-5–1* were located in the cytoplasm and extracellular stroma/chloroplast, respectively. Overexpression of *DIGLP1-5–1* in the globular embryos (GEs) of longan promoted the accumulation of lignin and decreased the H_2_O_2_ content by regulating the activities of ROS-related enzymes. The results provide a reference for the functional analysis of *DlGLPs* and related research on improving lignin accumulation in the agricultural industry through genetic engineering.

**Supplementary Information:**

The online version contains supplementary material available at 10.1186/s12864-023-09201-y.

## Introduction

Germin-like proteins (GLPs) and germin (GER) have long been recognized as marker proteins of the plant germination process. GLPs contain three typical conserved motifs: Box A [QDFCVAD], Box B [G(x)5Hx H(x)3,4E(x)6G] and Box C [G(x)5Px G(x) 2H(x)3N]. The three histidine residues and one glutamic acid residue in Box B and Box C may be involved in metal ion binding [1–3]. Based on the evolutionary relationships of the sequences, GLPs can be divided into six subfamilies: germinin-like subfamilies I, II and III, the true germinin subfamily, the gymnosperm-like germin subfamily and the moss-like germin subfamily [4]. To date, GLP genes have been widely studied in plants. A total of 32, 44, 20, 21, 7, 38 and 14 GLP family members were found in Arabidopsis (*Arabidopsis thaliana*) [5], banana (*Musa nana*) [6], grain (*Setaria italica*) [7], soybean (*Glycine max (Linn.)* [8], grape (*Vitis vinifera*) [9], cucumber (*Cucumis sativus*) [10] and rape (*Brassica napus*) [11], respectively.

GLPs are widely expressed in different organs, such as roots, stems, leaves, flowers and seeds; they also have diverse functions in different plant developmental stages [12, 13] and are mainly involved in a variety of physiological processes in the form of receptors, enzymes and structural proteins [14]. Previous studies have shown that *PsGLP1* and *PsGLP2* are involved in the whole process of flower and fruit development in different varieties of plum (*Prunus*) [15]. *OsGLP2-1* affects seed germination in rice (*Oryza sativa* L.) by participating in the abscisic acid (ABA) and gibberellin (GA) signaling pathways [16]. GLPs, arabinogalactan protein (AGP), chitinase (CHI), lipid transfer protein (LTP) and glycoprotein are key factors involved in plant somatic embryogenesis (SE) and are marker proteins during plant early SE [17]. Silencing of the *LmGLP* gene inhibited callus maturation in larch (*Larix x marschlinsii Coaz*) [18]. Overexpression and knockout of *OsGLP1* in rice calli did not affect the germination of young plants, but in the 6th month of the T0 generation, the mature old leaves showed anaphylactic necrosis [19, 20]. This result suggests that *GLP* genes play different roles during embryo developmental processes in different plants.

GLPs play roles in the response to biotic and abiotic stresses [21, 22]. For example, in the process of plant infection by pathogenic fungi, barley (*Hordeum vulgare* L.) *HvGLP4*, wheat (*Triticum aestivum* L.) *TaGLP4*, and rice (*Oryza sativa* L.) *OsRGLP2* and *OsGLP1* play similar roles as superoxide dismutase (SOD) and polyphenol oxidase in the apoplast. Hydrogen peroxide (H_2_O_2_) accumulates during reactive oxygen species (ROS)-induced pathogenic bacterial invasion, thus activating specific genes of the H_2_O_2_-mediated defense response [21, 23, 24]. Land cotton (*Gossypium hirsutum*) *GhGLP2* and growth hormone binding protein 19 (*G. hirsutum* auxin binding protein19, GhABP19) also possess SOD activity and are involved in vascular bundle browning and oxygen stress tolerance in response to pathogenic bacterial infestation [25, 26]. GLPs also exhibit oxalate oxidase (OXO) activity in both litchi and longan [27]. The *GLP* expression level in the carpopodium of 'Shi Xia' longan was negatively correlated with OXO activity [27]. Therefore, studying the role of *GLP* genes can provide further insights into their functions during early SE in longan.

As one of the most abundant biological materials on earth [28], lignin has been widely used in many fields as a reinforcing agent, binder and corrosion inhibitor [29, 30]. Lignin is mainly derived from plants in nature. Lignin also plays an important role in plants, affecting the formation of cell walls. Previous studies have shown that GLP genes are involved in cell wall formation and are enriched in the metabolic pathway of phenylpropane biosynthesis [31]. It is speculated that the DlGLP gene is involved in lignin biosynthesis. Therefore, studying the role of GLPs in plants is of great importance for obtaining plants with high wood quality in the agricultural industry.

Longan belongs to the Sapindaceae family and is an economically important fruit tree in the subtropical zone. Its fruit has high medicinal value, with the ability to delay aging and inhibit cancer cell growth. Embryo development has an important effect on fruit quality. Longan embryogenesis plays an extremely important role in fruit development, but it is a complex process that is influenced by various factors [32]. The metabolic synthesis of lignin is an important link during the SE of longan, which will affect the structural changes in the cell wall and the process of cell wall thickening during embryonic development [33]. The synthesis of lignin is affected by the activities of SOD, peroxidase (POD), catalase (CAT) and other enzymes. POD can produce phenoxy radicals by decomposing H_2_O_2_, which is the raw material for lignin synthesis [34]. Previous studies have shown that DNA replication, cell cycle-related genes, and the extracellular protein-encoding genes LTP, CHI, GLP, and AGP were significantly differentially expressed during early SE in longan, and they may be involved in regulating SE in longan [35]. The GLP gene acts as an SOD or OXO to regulate the H_2_O_2_ content in the process of plant growth and development, thus affecting the cross-linking of the cell wall [36]. Therefore, we speculate that the GLP gene affects lignin synthesis by regulating the ROS content in plants, thus participating in the growth and development of longan at the somatic embryo stage. In this study, we performed genome-wide identification of longan GLP (*DlGLP*) family members based on a longan genome and transcriptome dataset. The basic physicochemical properties, chromosomal distribution, conserved motifs, gene structure, evolutionary relationships, *cis*-acting elements, and transcriptome data of *DlGLPs* were investigated to determine the possible roles of *DlGLPs* in longan. We also investigated the expression patterns of *GLPs* during the early stage of SE in longan and under ABA and methyl jasmonate (MeJA) hormone treatments. The subcellular localization of the differentially expressed genes *DlGLP5-8–2* and *DlGLP1-5–1* was further verified. Overexpression of *DlGLP1-5–1* might increase the lignin content of longan globular embryos by dynamically regulating the ROS level in plants through SOD, POD and CAT activities. This study provides useful information for further research on the functional mechanisms of *GLP* genes in longan and provides a reference for improving lignin accumulation through genetic engineering in the agricultural industry.

## Materials and methods

### Materials

The materials for this experiment were 'Honghezi' longan embryogenic calli (ECs) preserved at the Institute of Horticultural Biotechnology, Fujian Agriculture and Forestry University. The ECs, incomplete compact proembryogenic cultures (ICpECs) and globular embryos (GEs) were obtained by the method described by Zhongxiong Lai et al. [37, 38] ECs (0.2 g) were cultured on MS liquid medium treated with ABA and MeJA at different concentrations (50 μmol/L, 100 μmol/L, 200 μmol/L) at 25 ℃ with 120 r·min^−1^ shaking in the dark for 24 h. The control group ECs were cultured on MS liquid medium alone, without ABA and MeJA treatment, under the same conditions. Each treatment was performed with three biological replicates, and the samples were collected and frozen in liquid nitrogen for standby.

### Identification of longan GLP family members and analysis of their protein physicochemical properties

The *A. thaliana GLP* gene sequence was obtained from The Arabidopsis Information Resource (TAIR; http://www.arabido-psis.org). The Arabidopsis GLP amino acid sequence was used as the probe sequence, and a single Blast screening of the longan genome database (BioProject accession: PRJNA792504; genome sequence of *D. longan*: SRR17675476) and TAIR (https://www.arabidopsis.org/) was performed using TBtools software [39], followed by another two-way Blast screening against the National Center for Biotechnology Information (NCBI) database ([Internet]. Bethesda (MD): National Library of Medicine (US), National Center for Biotechnology Information; [1988] – [cited 2022 Sep 24]).(https://www.ncbi.nlm.nih.gov/geo/) to identify candidate DlGLP members. The conserved structural domains of the candidate sequences were analyzed by HMMER online software (https://www.ebi.ac.uk/Tools/hmmer/search/phmmer), and a total of 35 DlGLP members with a complete conserved structural domain (Cupin-1) were obtained and named according to the results of homology comparison with Arabidopsis [40]. The amino acid sequences of DlGLPs were submitted to the ExPASy (http://au.expasy.org/) website for the analysis of physicochemical properties, including amino acid number, instability coefficient, isoelectric point (pI), molecular mass, and hydrophilicity [41]. The signal peptides of the DlGLP family were predicted using the SignalP4.0 (http://www.cbs.dtu.dk/services/SignalP/) website, and their predicted subcellular localization was determined via the online website WoLF PSORT (https://wolfpsort.hgc.jp/) [42]. Prediction of the DlGLP1-5–1 and DlGLP5-8–2 tertiary structures was performed using the SWISS-MODEL (https://swissmodel.expasy.org/) website [43].

### Construction of the phylogenetic tree of DlGLP family members

MEGA-X 10.0.5 software was used to conduct multiple sequence alignment of amino acid sequences, and the maximum likelihood method was used to construct the evolutionary tree of DlGLP family members. The amino acid sequences with large homologous differences were manually deleted, and the bootstrap coefficient was set to 1000 repeated tests [44]. By this method, phylogenetic trees of GLP family members of six species, namely, longan, Arabidopsis, lychee [45], rice, banana and grape [9], were constructed. The online tool iTOL (https://itol.embl.de/upload.cgi) was used to visualize the evolutionary tree [46].

### Gene structure, chromosomal localization, and covariance analysis of DlGLP family members

Gene structure analysis of DlGLP family members was performed using gene annotation files from the longan database and TBtools software; conserved structural domain information was obtained from the NCBI (https://www.ncbi.nlm.nih.gov/geo/) website; MEME (http://meme-suite.org/) online software was used for DlGLP family protein motif analysis; and visualization was performed with TBtools software [47]. Chromosomal localization and covariance analysis of DlGLP family members were performed using gene annotation files from the longan database and TBtools software, and the results were visualized using TBtools software.

### Analysis of *cis-*acting elements of the DlGLP family

The sequences 2000 bp upstream of the translation start codon of DlGLP family members were extracted as the promoter sequences of DlGLPs by TBtools software using the gene structure annotation file of the Longan Genome Database (NCBI Registration Number: PRJNA792504), and the data were analyzed by using the PlantCARE (http://bioinformatics.psb.ugent.be/webtools/plantcare/html/) website for identification of *cis*-acting elements in the promoters [48]. TBtools software was used for visualization. The transcription factors regulating the target genes were predicted using the PlantTFDB (http://planttfdb.gao-lab.org/index.php) website [49].

### Transcriptomic analysis of DlGLP at early stages of SE, at different tissue sites, at different temperatures and under SL treatment

The FPKMs of the DlGLP gene family were analyzed using longan transcriptome datasets generated from the early stage of SE (ECs, ICpECs and GEs) in 'Honghezi' longan (NCBI BioProject number: PRJNA891444) and nine different tissues and organs of 'SJM' longan (NCBI BioProject number: PRJNA326792) [50], and ECs treated with 1 mol·L^−1^ 2,4-D for 4 h, 8 h and 24 h (NCBI BioProject number: PRJNA889670), different temperatures (15 ℃, 25 ℃ and 35 ℃) (NCBI BioProject number: PRJNA889670), and 1 μmol·L^−1^ strigolactone (SL) (NCBI BioProject number: PRJNA889220) treatment for 4 h. The FPKM values of 35 DlGLPs extracted from different longan transcriptome databases were analyzed by log2 analysis, a heatmap was drawn by TBtools software, and cluster analysis was conducted.

### qRT‒PCR validation of DlGLPs at the early stage of somatic embryogenesis and under ABA and MeJA treatments

RNA was extracted using the TransZol Up Kit (All Style Gold, Beijing, China) according to the manufacturer’s instructions. cDNA synthesis was performed using PrimeScriptTM IV 1st Strand cDNA Synthesis Mix (Bao Ri Medical, Beijing). Primer design was performed using DNAMAN 6.0 software (Table S1). Quantitative real-time polymerase chain reaction (qRT‒PCR) amplification was conducted using Hieff qPCR SYBR Green Master Mix (Solabao, Beijing) in a LightCycler480 instrument (Roche Diagnostics). The 20 μL reaction system contained the following: SYBR Premix Ex Taq TM II (Bao Ri Medical, Beijing, China), 10 μL; ddH_2_O, 6.4 μL; upstream and downstream primers, 0.8 μL each; cDNA template, 2 μL. *EUKARYOTIC ELONGATION FACTOR 1-ALPHA* (EF-1ɑ) was used as the internal reference gene during early SE in longan, and *UBIQUITIN* (UBQ) was used as the internal reference gene quantified under different hormone treatments. The data were calculated according to the 2^−ΔΔCt^ method, three technical replicates were set for each sample, and three biological replicates were set for each gene. SPSS 20 software was used for data analysis [51]. The correlation between relative expression amount and FPKM value was mapped with Prism 8.0.2 software. In all graphs, error bars indicate standard deviation, and significant differences are indicated with “*” (*p* < 0.05), “**” (*p* < 0.01), “***” (*p* < 0.005), or “****” (*p* < 0.001) using Student’s t test.

### Subcellular localization analysis

The full-length coding sequence of the DlGLP gene (*DlGLP1-5–1/5–8-2*) without a termination codon was amplified with primers (Table S2) and cloned into the pCAMBIA1302-35S-GFP vector. The bacterial solution containing the recombinant plasmid was activated and centrifuged at 5000 r/min for 10 min to collect the cell pellet. MS penetrant (3% sucrose, 50 µmol·L^−1^ acetylsyringone (AS) and 10 µmol·L^−1^ MgCl_2_) was used to resuspend the cell pellet, and the OD_600_ was adjusted to 0.6–0.9. The prepared infection solution was injected into the lower epidermis of tobacco leaves and cultured at 25 °C darkness for three days. The lower epidermis of the tobacco leaves was disrupted by the GFP fusion protein expression method. The cells were placed under a laser confocal microscope (Olympus FV 1000) for observation.

### Stable transformation of longan ECs and determination of their physiological indexes

The full-length coding sequence of the *DlGLP1-5–1* gene was amplified with primers (Table S2) and cloned into the pCAMBIA1301-35S-GUS vector. The pCAMBIA1301-35S-*DlGLP1-5–*1: GUS construct was transformed into *Agrobacterium tumefaciens* (GV3101). The rapidly proliferating longan ECs were transferred to the culture of Agrobacterium cells containing the recombinant plasmid (OD600 = 0.6–0.8). After 30 min of infection, the ECs were transferred to MS solid medium containing 30 g/L sucrose for 3 days. Then, the cells were transferred to MS solid medium containing 1.0 mg/L 2,4-D, 20 g/L sucrose and 300 mg/L cefotaxime for 20 days. The proliferated ECs were transferred to MS solid selective medium containing 1.0 mg/L 2,4-D, 20 g/L sucrose, 300 mg/L cefotaxime and 20 mg/L hygromycin. They were subcultured once a month until positive materials were obtained. GUS staining (Beijing Huayueyang Biotechnology Co., Ltd., China) and PCR amplification was used to detect transformed GEs. The materials with good growth conditions after subculture for 20 days were selected to test the activities of SOD, POD and CAT and the levels of lignin and H_2_O_2_. The enzyme activities of SOD, POD and CAT were measured with the corresponding kits (Solarbio, Beijing, China). The lignin content in longan GEs was measured with a lignin detection kit (Solarbio, Beijing, China). Please refer to the manufacturer’s instructions for specific methods.

## Results

### Identification of the DlGLP family and analysis of protein physicochemical properties

**Fig. 1:**
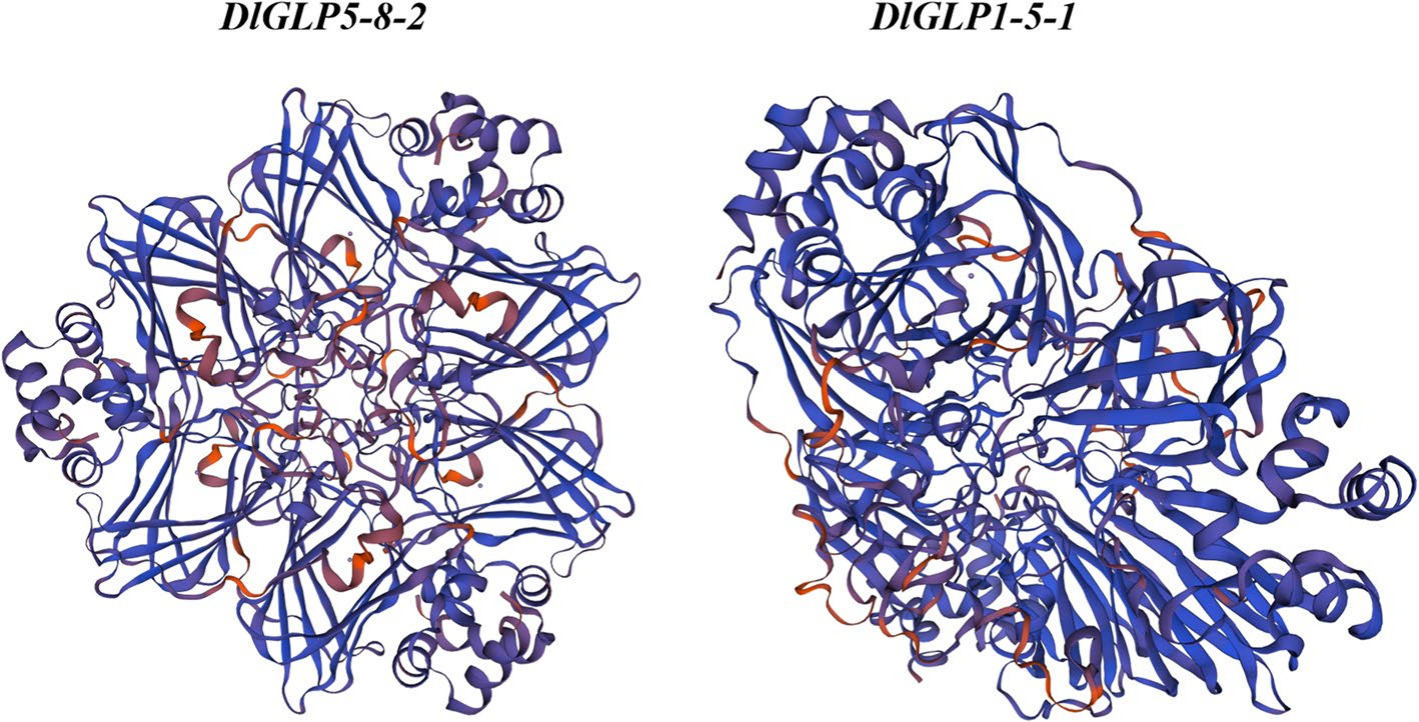
Tertiary structure of DlGLP1-5–1 and DlGLP5-8–2

A total of 35 *DlGLP* gene family members containing the complete conserved structural domain (Cupin-1) were screened from the third-generation longan genome. The protein length of DlGLP family members ranged from 203–239 amino acids (Table 1); the molecular weight ranged from 21.43–26.14 Da, and the pI ranged from 5.32–9.37. The subcellular localization prediction results showed that most of the DlGLP family members were localized in the extracellular matrix. The α-helix proportion of the 35 DlGLP members ranged from 15 to 30%. Most DlGLP members had β-helix angles less than 10%, and only *DlGLP5-8–3* had β-helical angles greater than 10%. (Table 1). Except for *DlGLP3-8–*1, all of the DlGLPs contained signal peptides. Tertiary structure prediction suggested that both DlGLP1-5–1 and DlGLP5-8–2 had OXO activity (Fig. 1) [52].

**Table 1:** The physicochemical properties of the proteins of longan GLP family member

Third generation gene ID	Second generation gene ID	Gene name	Corresponding Arabidopsis name	Corresponding Arabidopsis ID	Protein length (aa)	Molecular weight/Da	PI	Instability coefficient	Fat coefficient	Hydrophilia	α- helix	β- corner	Extension chain	Irregular curl	Subcellular localization	Signal peptide
Dlo034548	Dlo_025479.1	*DlGLP3-8–2*	*AtGLP3-8*	AT3G10080	211	22,961.57	7.74	26.53	97.82	0.143	0.2227	0.0758	0.2701	0.4313	Chloroplast	Yes
Dlo027099	Dlo_030033.1	*DlGLP5-14*	*AtGLP5-14*	AT5G39190	216	22,938.49	9.05	25.27	100.23	0.301	0.2407	0.0787	0.287	0.3935	Extracell	Yes
Dlo005984	Dlo_010246.1	*DlGLP5-1–1*	*AtGLP5-1*	AT5G20630	218	22,973.75	9.37	15.33	101.56	0.234	0.2431	0.0734	0.2661	0.4174	Extracell	Yes
Dlo004158	Dlo_014585.1	*DlGLP5-7–2*	*AtGLP5-7*	AT5G39100	225	24,331.18	8.53	23.38	96.18	0.161	0.2357	0.0684	0.2738	0.4221	Cytoplasm	Yes
Dlo027098	Dlo_030033.1	*DlGLP5-7–6*	*AtGLP5-7*	AT5G39100	220	23,490.24	9.07	23.99	99.27	0.314	0.2318	0.0682	0.2864	0.4136	Extracell	Yes
Dlo004157	Dlo_014586.1	*DlGLP5-7–4*	*AtGLP5-7*	AT5G39100	222	23,928.48	6.39	30.97	99.64	0.265	0.2432	0.0766	0.2928	0.3874	Extracell	Yes
Dlo004174	Dlo_014569.1	*DlGLP5-8–5*	*AtGLP5-8*	AT5G39110	225	24,128.67	7.78	25.32	98.76	0.191	0.2311	0.0622	0.2844	0.4222	Cytoplasm	Yes
Dlo004173	Dlo_014569.1	*DlGLP5-8–4*	*AtGLP5-8*	AT5G39110	225	24,142.74	8.52	25.32	98.76	0.189	0.2222	0.0933	0.2889	0.3956	Extracell	Yes
Dlo000252	Dlo_004986.1	*DlGLP5-1–2*	*AtGLP5-1*	AT5G20630	208	21,431.79	5.83	39.45	99.9	0.531	0.2308	0.0769	0.2885	0.4038	Vacuole	Yes
Dlo004176	Dlo_014566.1	*DlGLP5-8–6*	*AtGLP5-8*	AT5G39110	225	24,193.79	7.8	25.98	100.04	0.212	0.24	0.0711	0.2756	0.4133	Cytoplasm	Yes
Dlo029651	Dlo_009699.1	*DlGLP3-8–1*	*AtGLP3-8*	AT3G10080	203	21,930.09	8.95	24.88	79.16	-0.063	0.1576	0.069	0.3005	0.4729	Nucleus	No
Dlo004175	Dlo_014567.1	*DlGLP5-8–3*	*AtGLP5-8*	AT5G39110	224	24,114.45	6.81	23.27	87.1	0.074	0.2188	0.1071	0.2768	0.3973	Extracell	Yes
Dlo006198	Dlo_004986.1	*DlGLP1-6*	*AtGLP1-6*	AT1G72610	209	21,758.35	9.1	23.27	106.89	0.494	0.1914	0.0718	0.2823	0.4545	Extracell	Yes
Dlo025974	Dlo_032498.1	*DlGLP1-1*	*AtGLP1-1*	AT1G02335	207	22,020.1	5.22	27.63	84.88	0.2	0.2319	0.058	0.2609	0.4493	Extracell	Yes
Dlo025972	Dlo_032496.1	*DlGLP3-9–2*	*AtGLP3-9*	AT3G62020	206	21,654.92	5.83	23.51	92.43	0.283	0.2524	0.0825	0.2524	0.4126	Extracell	Yes
Dlo025973	Dlo_032497.1	*DlGLP3-9–3*	*AtGLP3-9*	AT3G62020	210	22,005.34	6.82	24.55	96.19	0.392	0.1905	0.0714	0.3238	0.4143	Extracell	Yes
Dlo004159	Dlo_014582.1	*DlGLP5-11*	*AtGLP5-11*	AT5G39150	224	24,129.72	6.81	22.8	93.17	0.115	0.2411	0.0982	0.2902	0.3705	Extracell	Yes
Dlo034709	Dlo_025473.1	*DlGLP3-8–4*	*AtGLP3-8*	AT3G10080	212	23,117.75	7.01	29.57	97.78	0.192	0.217	0.066	0.2877	0.4292	Chloroplast	Yes
Dlo013521	Dlo_025831.1	*DlGLP5-7–7*	*AtGLP5-7*	AT5G39100	213	22,436.92	6.94	36.86	92.86	0.192	0.2347	0.0657	0.2582	0.4413	Extracell	Yes
Dlo004160	Dlo_014581.1	*DlGLP5-8–1*	*AtGLP5-8*	AT5G39110	224	24,197.62	6.27	25.58	89.6	0.084	0.2188	0.0759	0.2679	0.4375	Endoplasmic reticulum	Yes
Dlo018636	Dlo_000721.1	*DlGLP1-3*	*AtGLP1-3*	AT1G10460	218	23,187.57	6.39	28.79	96.24	0.183	0.2477	0.0826	0.2523	0.4174	Extracell	Yes
Dlo011500	Dlo_025475.1	*DlGLP3-8–6*	*AtGLP3-8*	AT3G10080	209	22,830.43	7.01	28.93	97.32	0.184	0.2297	0.067	0.2967	0.4067	Chloroplast/Mitochondrion	Yes
Dlo027100	Dlo_030033.1	*DlGLP5-7–3*	*AtGLP5-7*	AT5G39100	216	23,139.76	9.05	29.4	102.92	0.301	0.2361	0.0602	0.2778	0.4259	Peroxisome	Yes
Dlo004166	Dlo_014572.1	*DlGLP5-12*	*AtGLP5-12*	AT5G39160	239	26,103.73	6.89	24.62	84.44	-0.012	0.1883	0.0753	0.3305	0.4059	Extracell	Yes
Dlo004169	Dlo_014572.1	*DlGLP5-8–7*	*AtGLP5-8*	AT5G39110	239	26,132.68	6.89	23.23	82.8	-0.045	0.1799	0.0711	0.2427	0.5063	Extracell	Yes
Dlo022073	Dlo_020307.1	*DlGLP3-7*	*AtGLP3-7*	AT5G39100	215	22,884.45	8.45	31.4	104.79	0.21	0.2093	0.0744	0.2977	0.4186	Extracelll	Yes
Dlo004163	Dlo_014582.1	*DlGLP5-7–1*	*AtGLP5-7*	AT5G39100	214	22,626.07	6.81	23.92	96.54	0.224	0.1729	0.0935	0.2897	0.4439	Extracell	Yes
Dlo004162	Dlo_014583.1	*DlGLP5-7–5*	*AtGLP5-7*	AT5G39100	225	24,004.72	7.76	30.23	99.69	0.228	0.2578	0.0756	0.2756	0.3911	Extracell	Yes
Dlo004164	Dlo_014581.1	*DlGLP5-8–2*	*AtGLP5-8*	AT5G39110	224	24,234.64	5.88	23.58	90.04	0.028	0.1607	0.0848	0.2857	0.4688	Cytoplasm	Yes
Dlo032152	Dlo_012402.1	*DlGLP1-5–1*	*AtGLP1-5*	AT1G18980	221	22,977.44	7.69	16.62	99.68	0.397	0.1765	0.0769	0.2896	0.457	Extracell/Chloroplast	Yes
Dlo034551	Dlo_025475.1	*DlGLP3-8–5*	*AtGLP3-8*	AT3G10080	209	22,858.44	6.59	30.9	97.32	0.17	0.2249	0.0813	0.2919	0.4019	Chloroplast	Yes
Dlo034550	Dlo_025473.1	*DlGLP3-8–3*	*AtGLP3-8*	AT3G10080	212	23,159.83	7.01	30.96	98.25	0.175	0.217	0.066	0.2689	0.4481	Mitochondrion	Yes
Dlo032150	Dlo_012401.1	*DlGLP1-5–2*	*AtGLP1-5*	AT1G18980	221	23,036.34	6.03	19.16	93.53	0.383	0.1674	0.0679	0.3167	0.448	Extracell	Yes
Dlo004171	Dlo_014572.1	*DlGLP5-8–8*	*AtGLP5-8*	AT5G39110	239	26,132.68	6.89	23.23	82.8	-0.045	0.1799	0.0711	0.2427	0.5063	Extracell	Yes
Dlo011877	Dlo_026676.1	*DlGLP3-9–1*	*AtGLP3-9*	AT3G62020	217	23,177.65	8.46	21.23	95.71	0.094	0.2166	0.0553	0.2857	0.4424	Extracell	Yes

### Phylogenetic tree of DlGLP family members

**Fig. 2:**
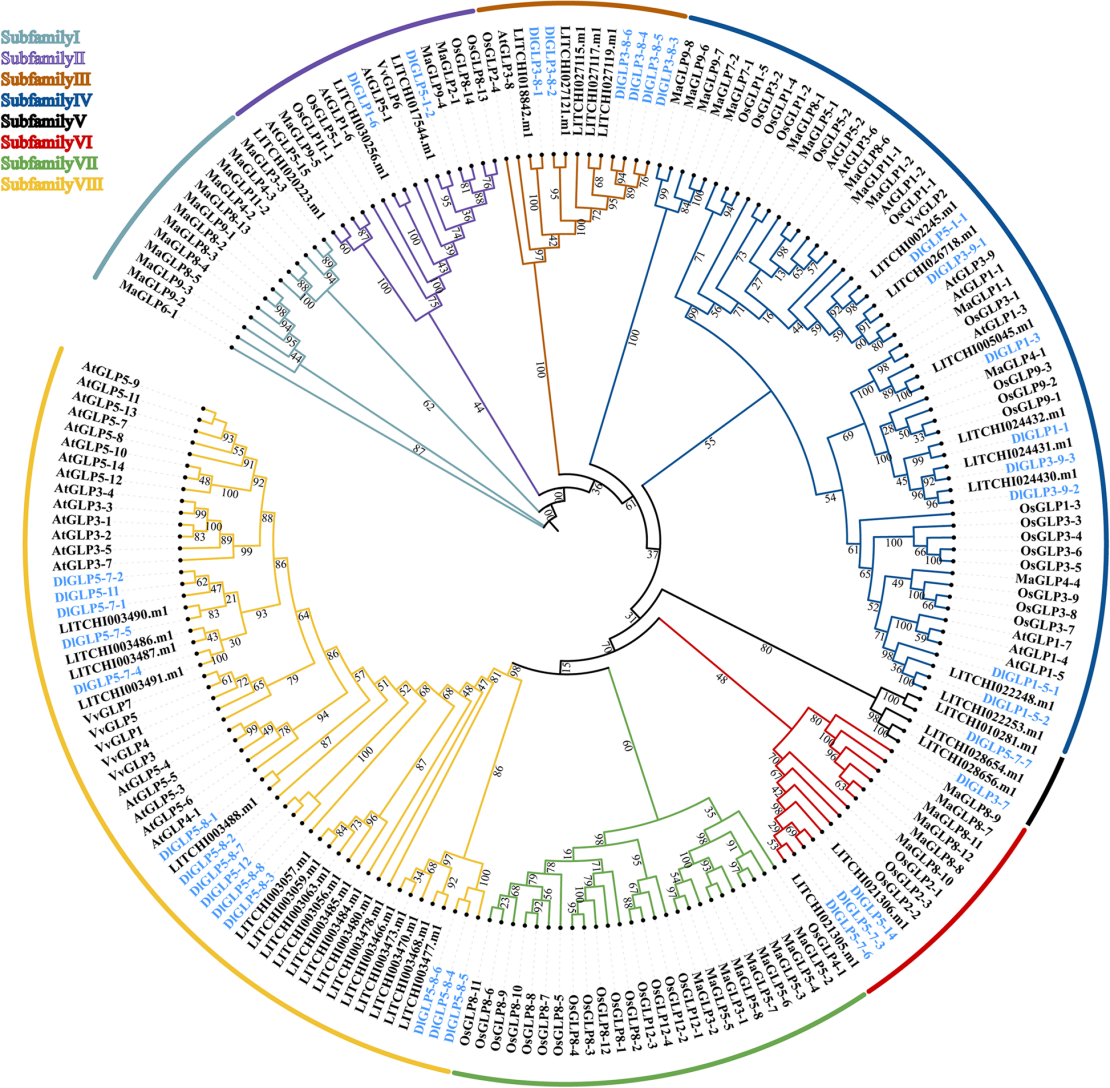
Evolutionary tree of GLP family members in Longan (Dl), Lychee (LTCHI), Arabidopsis (At), Rice (Os), Banana (Ma) and Grape (Vv)

To clarify the genetic relationship of DlGLPs, we constructed a phylogenetic tree of 200 GLP gene members, including longan (35 members), Arabidopsis (32 members), lychee (39 members), rice (43 members), banana (44 members), and grape (7 members) (Fig. 2). The GLP genes were classified into eight subfamilies, namely, subfamilies I-VIII. Among the eight subfamilies, the first subfamily contains only banana GLP genes, and these eight banana GLP genes possess multiple conserved cupin-1 domains. GLPs in the same species showed a large amount of aggregation in the fourth, seventh and eighth subgroups. This suggests that the GLP gene is conserved. Only lychee and longan GLP genes were found in the fifth subgroup, suggesting that GLP genes with unique functions may have evolved in Sapindaceae.

### Analysis of gene structure, chromosomal localization and collinearity of DlGLP family members

**Fig. 3:**
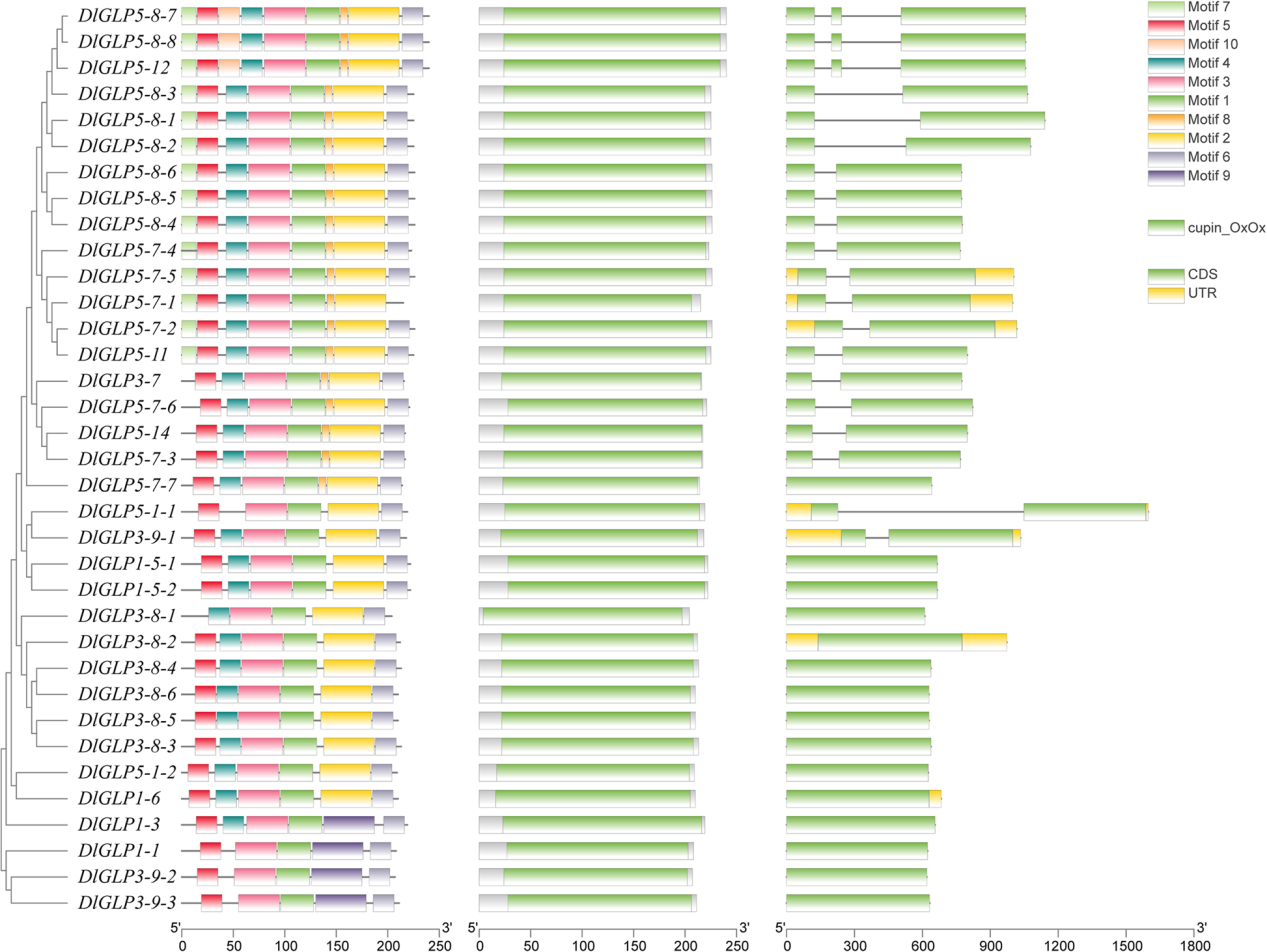
Conserved motif distribution and gene structure of DlGLP family members

The conserved motif and gene structure analysis results showed that the conserved motif number of each DlGLP ranged from 5 to 10, and the intron number ranged from 0 to 3 (Fig. 3). All of the DlGLPs contained motif 1 and motif 3; moreover, all the members contained motifs 5 and 6 with the exception of *DlGLP5-7–1* (lacked motif 6) and *DlGLP3-8–1* (lacked motif 5). These findings combined with signal peptide analysis showed that DlGLP3-8–1 was the only DlGLP family member that did not have motif 5. This suggests that motif 5 may be closely related to the formation of signal peptides. By a combination of gene structure and motif analysis, we found that the motif distribution and gene structure were relatively similar within the same subfamily. Li [53] found that the GLP genes in the same subfamily in rice and Arabidopsis had similar structures. In general, there were more conserved motifs and similar gene structures within the same subfamily of DlGLPs, which indicated that each subfamily of DlGLPs may perform distinct functions.

**Fig. 4:**
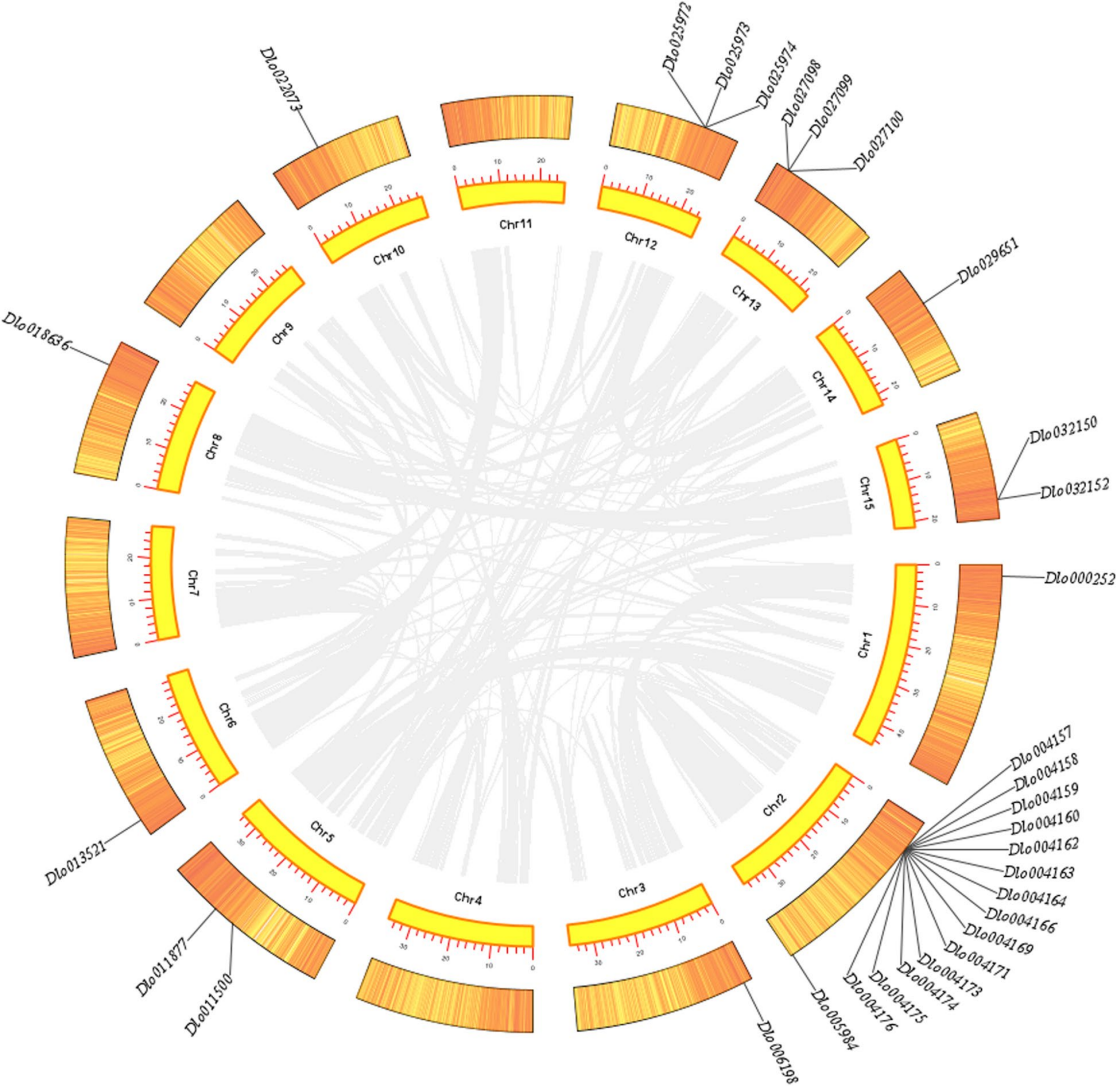
Chromosomal localization and covariance analysis of DlGLP family members. The gray lines represent the collinear blocks within the longan genome; Heat map represents gene density

Chromosomal localization results revealed that the 35 DlGLP genes were unevenly distributed on chromosomes 1–15 (Fig. 4). Gene duplication is one of the main drivers of genome evolution [54], and tandem duplication has also been suggested to be one of the main reasons for the expansion of plant gene families [55]. The main feature of tandem duplication is that multiple members of the family in question are clustered in the same or adjacent gene intervals [56]. Tandem duplication of the DlGLP gene was also found on chromosomes 2, 12, 13 and 15. Among them, 15 DlGLP genes were distributed on chromosome 2, and 14 of them were clustered. To further verify whether segmented duplication events occurred in DlGLP genes, we performed covariance analysis (Fig. 4), and the results showed that there was no covariance in DlGLP genes within the species, indicating that there were no segmented duplication events in DlGLP genes within the species. For longan, the number of GLP genes in tandem duplication regions was far greater than that in segmented duplication regions. Therefore, tandem duplication seems to play an important role in the expansion of the DlGLP family.

### *Predictive analysis of* cis*-acting elements of DlGLP family members*

**Fig. 5:**
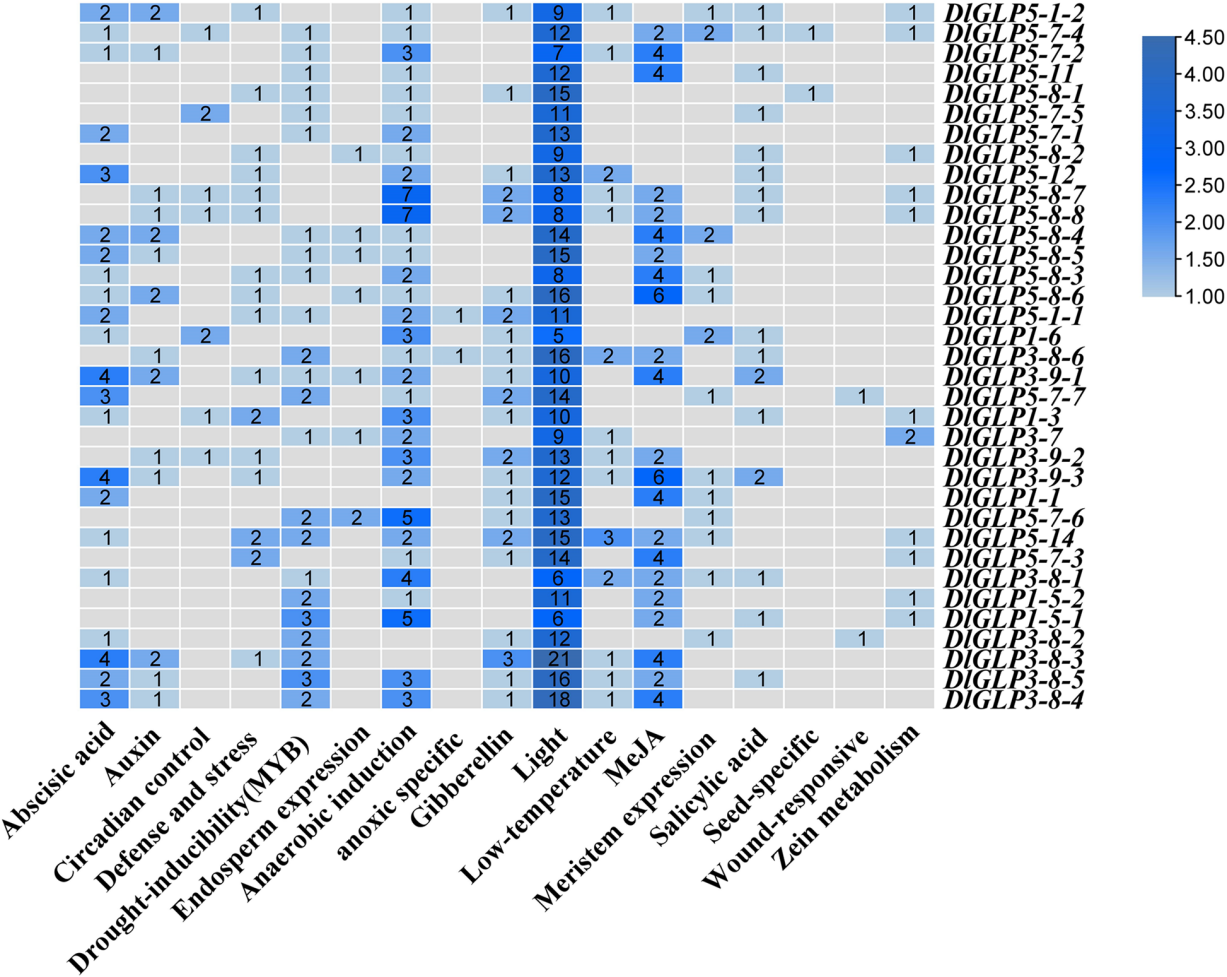
Distribution of *cis-*acting elements of DlGLP family members

To further explore the functions of DlGLPs in longan, the *cis*-acting elements localized upstream of the translation initiation site were predicted using the PlantCARE online tool [57]. The results indicated that the DlGLP promoter regions contained multiple growth and development-, hormone-, and stress-related response elements (Fig. 5). MeJA and ABA response elements accounted for a very high proportion of hormone response elements, with 70 and 44 of these present, respectively, suggesting that DlGLP genes may play an important role in the response to MeJA and ABA. Except for *DlGLP1-1*, *DlGLP3-8–2*, and *DlGLP3-8–3*, all of the DlGLP genes contained at least one anaerobicity-inducible element. Drought-inducible elements (35) were also widely distributed in the promoter regions of the DlGLP genes. These results suggested that DlGLP genes play a wide range of roles in the response to hormones, drought stress and anaerobicity-induced adversity in longan.

### RNA-seq revealed the expression profiles of longan DlGLP family genes in different tissues and treatments

To further investigate the expression patterns of the GLP family members in longan, the FPKM values of DlGLPs were extracted from the longan transcriptome database.

**Fig. 6:**
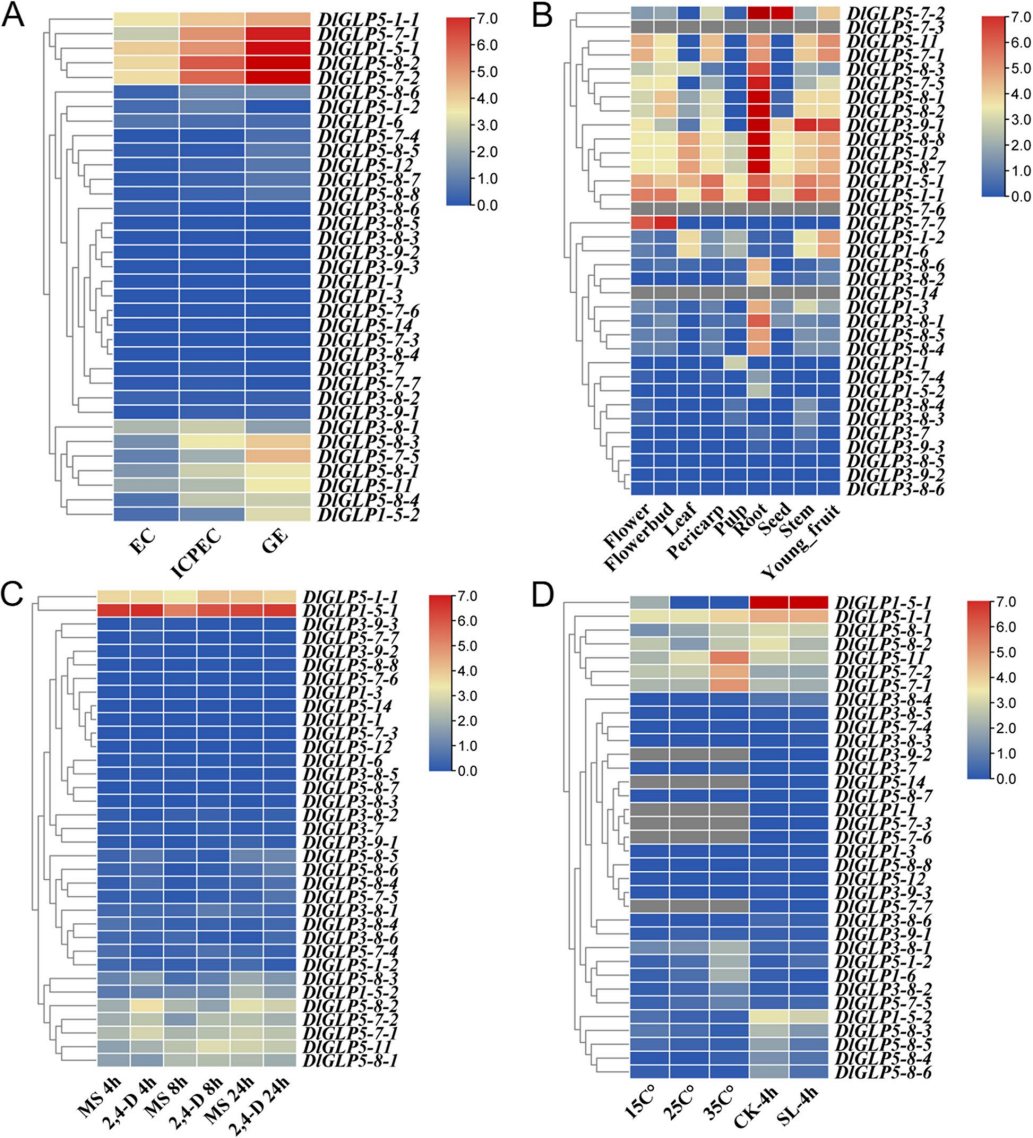
Expression profiles of DlGLP family genes in different tissues and treatments of longan. **A** Expression of *DlGLP* in different tissues of ‘SJM’ Longan. **B** Expression of *DlGLP* genes in early somatic embryogenesis in longan. **C** Expression of *DlGLP* genes in ECs under 2,4-D treatment. **D** Expression of *DlGLP* genes in ECs under temperature and SL treatments

The FPKM values of DlGLPs in nine different tissues and somatic embryos were extracted from the transcriptome for analysis. Transcriptome data from different tissues showed that most of the DlGLP genes were expressed in nine different tissue sites and were highly expressed in roots. *DlGLP5-1–1*, *DlGLP5-12*, *DlGLP5-8–7*, *DlGLP1-5–1*, and *DlGLP5-8–8* were highly expressed in all tissues (Fig. 6A), suggesting that they may be widely involved in the growth and developmental processes in longan. Sixteen DlGLP genes were upregulated during early SE in longan (Fig. 6B). Compared with the other DlGLPs, *DlGLP5-7–1*, *DlGLP1-5–1*, *DlGLP5-7–2* and *DlGLP5-8–2* showed higher expression levels with increasing expression trends during early SE in longan. These results suggested that DlGLPs were involved in the development of the GE stage. According to previous studies, GLPs are involved in the production of H_2_O_2_ during plant growth and development via SOD and OXO enzyme activities, which can strengthen the cell wall through cellulose cross-linking and are associated with cell wall formation as well as lignin biosynthesis [58]. Therefore, we speculated that DlGLPs may play an important role in the morphogenesis of the cell wall in the GE stage.

The transcriptome data of DlGLP genes under 2,4-D, SL and temperature treatments were further investigated. Most of the DlGLP gene family members showed upregulated expression in ECs under 2,4-D hormone treatment (Fig. 6C). The expression levels of *DlGLP1-5–1*, *DlGLP5-8–2*, and *DlGLP5-1–1* were significantly higher than those of other genes. The expression of *DlGLP1-5–1* under 2,4-D treatment was higher than that in the control group; *DlGLP5-8–2* showed a significant increase in expression at 4 h of 2,4-D treatment but showed a repressive effect at 8 h and 24 h; *DlGLP5-1–1* responded to 2,4-D regulation at 8 h only.

The expression levels of most DlGLP genes were downregulated in ECs under 1 μmol·L^−1^ SL treatment (Fig. 6D). The expression of *DlGLP1-5–1* and *DlGLP5-8–2* was also decreased, and these two genes may be involved in the SL signaling pathway. The expression of some DlGLP genes increased under high-temperature treatment (35℃), so these genes may play roles under high-temperature stress (Fig. 6D). However, only *DlGLP1-5–1* and *DlGLP5-8–2* showed significantly upregulated expression under low-temperature treatment (15 °C).

Through analysis of different transcriptomes, it was found that *DlGLP1-5–1*, *DlGLP5-8–2* and *DlGLP5-1–1* were highly expressed in each transcriptome, suggesting that these three genes are involved in the growth and development of longan somatic embryos. According to the evolutionary tree analysis, the three genes belonged to different subfamilies, and their expression patterns in the transcriptome were different, suggesting that the DlGLP genes function differently in different subfamilies.

### Analysis of the DlGLP expression patterns during early SE and under different hormone treatments in longan

**Fig. 7:**
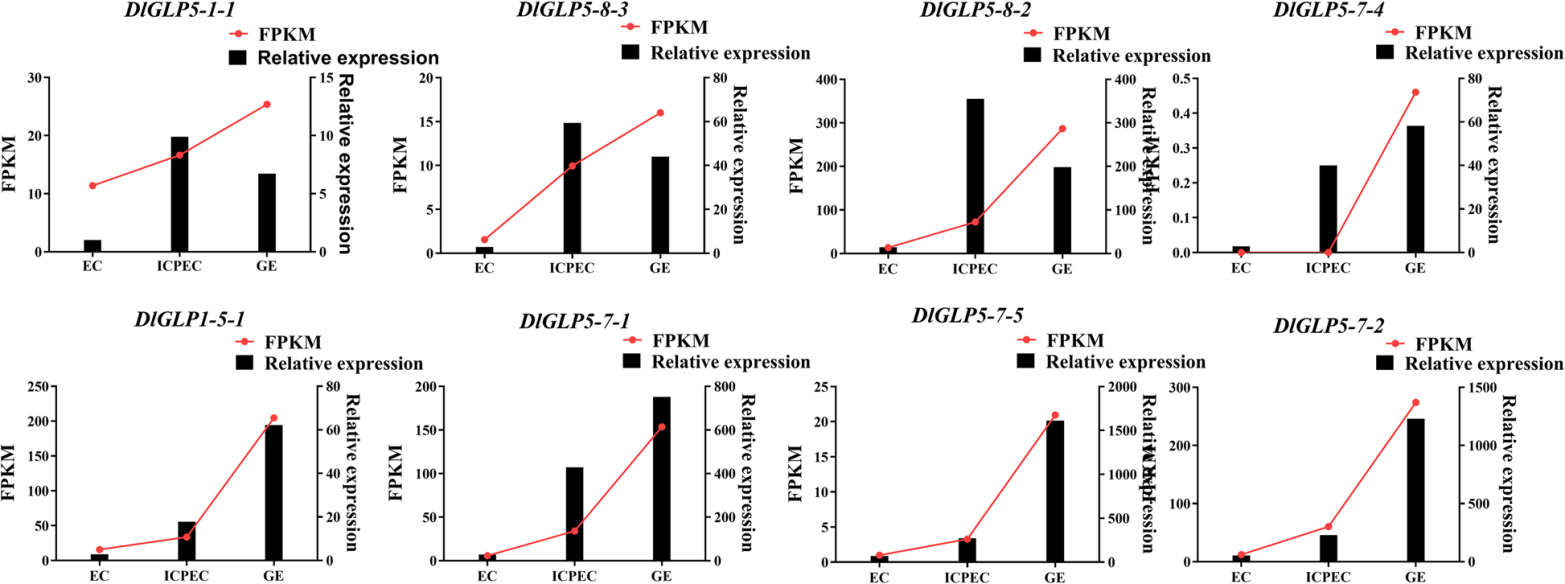
Relative expression of some members of the DlGLP family in the early stage of somatic embryogenesis in longan

qRT‒PCR was used to verify the reliability of the DlGLP expression patterns during early SE in longan. By analyzing the transcriptome data of longan somatic embryos, we further selected eight *DlGLP* genes that were highly expressed at the somatic embryo stage for qRT‒PCR assays. The results showed that five DlGLP genes (*DlGLP5-7–4*, *DlGLP1-5–1*, *DlGLP5-7–1*, *DlGLP5-7–5*, and *DlGLP5-7–2*) were upregulated during early SE in longan and had the same expression trends as those in the transcriptome dataset (Fig. 7), demonstrating that these five genes may play important roles in the development of the GE stage. *DlGLP5-1–1*, *DlGLP5-8–3*, and *DlGLP5-8–2* showed higher expression levels in the ICpEC stage than in the EC and GE stages, indicating that these three genes may play an important role in morphogenesis during early GE formation.

**Fig. 8:**
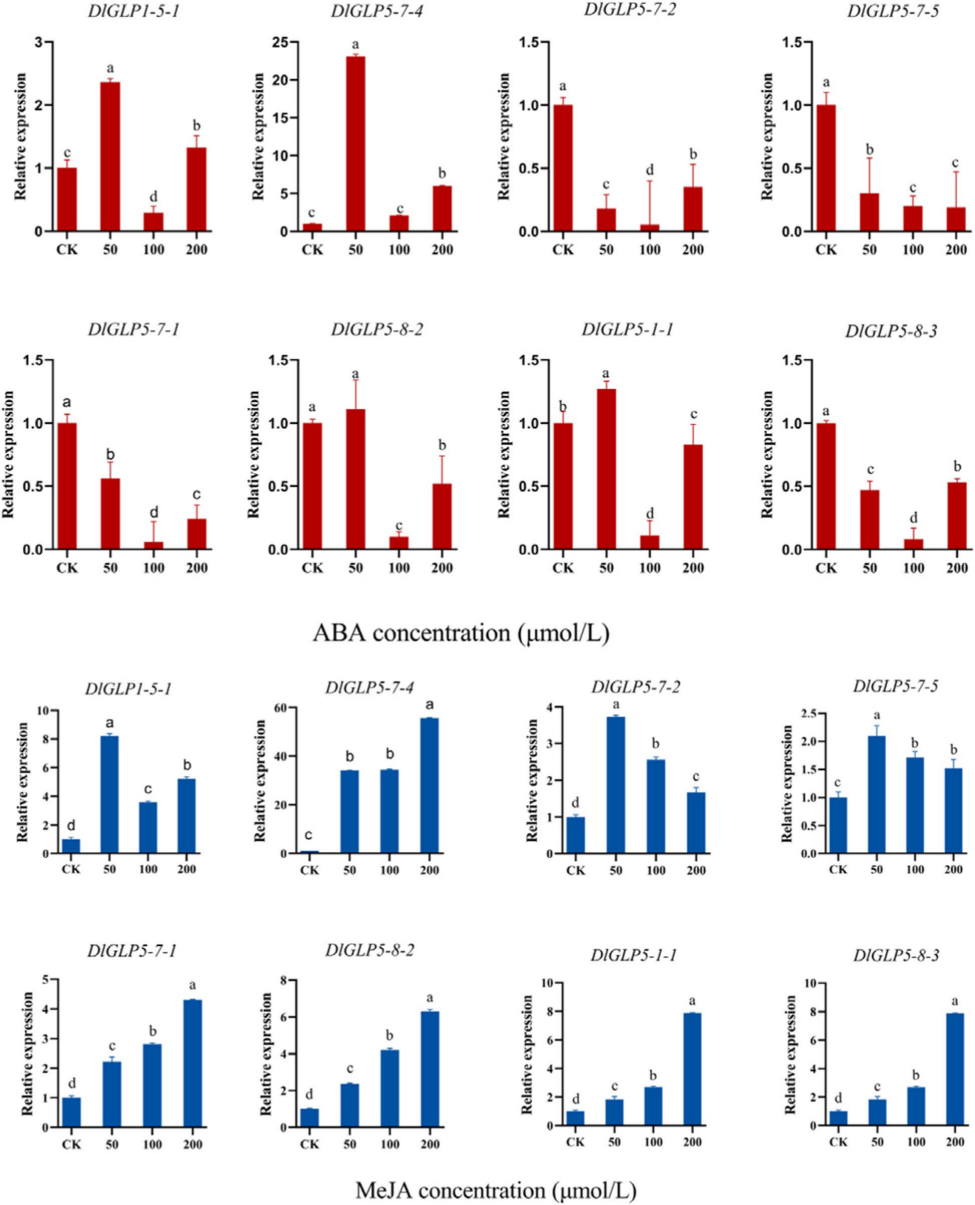
Expression levels of some members of the DlGLP family under abscisic acid (ABA), methyl jasmonate (MeJA), and clear water control (CK) treatments

Based on the results of *cis*-acting element analysis of the promoters of the DlGLP family, qRT‒PCR was used to detect the expression levels of eight DlGLP genes in longan ECs under two exogenous hormone (ABA and MeJA) treatments (Fig. 8). Four *DlGLP* genes were significantly downregulated under ABA treatment. Under treatment with 100 μmol·L^−1^ ABA, the expression of all seven DlGLP genes except DlGLP5-7–4 showed significant downregulation. Moreover, the expression of DlGLP5-7–4 was significantly upregulated when the ABA concentration was 50 μmol·L^−1^, indicating that DlGLP5-7–4 played an important role in the ABA signaling pathway during somatic embryo development in longan. The expression of DlGLPs was affected by changes in the ABA concentration. All eight DlGLP genes showed upregulated expression under MeJA treatment. This suggests that DlGLPs may play an important role in the MeJA signaling pathway. These results further indicate that different subfamilies of DlGLPs perform different functions during somatic embryo development in longan.

### Subcellular localization analysis

**Fig. 9:**
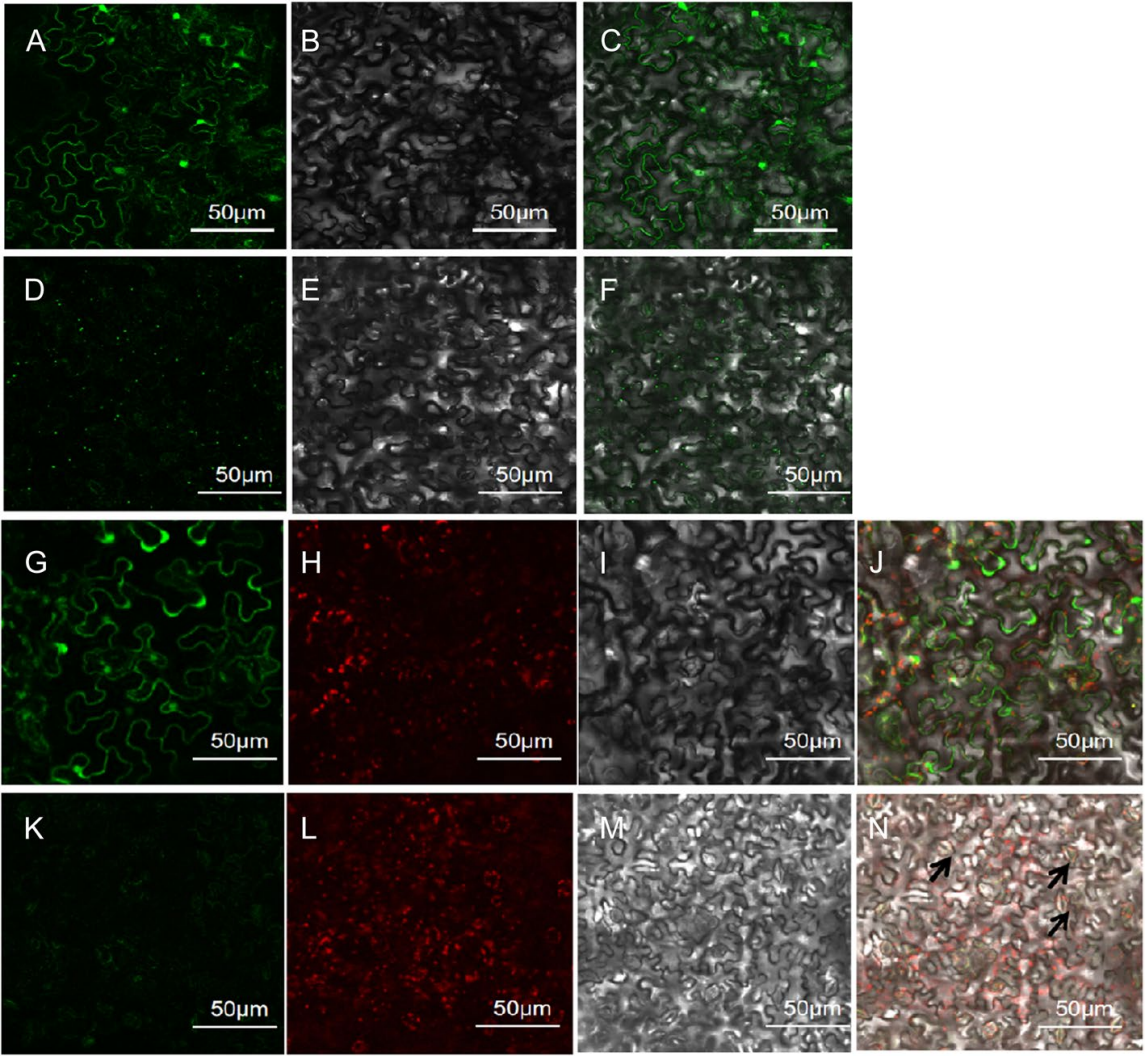
Subcellular localization of pCAMBIA1302-35S-GFP empty, *DlGLP5-8–2* and *DlGLP1-5–1* in tobacco. **A-C** Tobacco leaf subepidermal cells introduced with pCAMBIA1302-35S-GFP empty vector, **D**-**F**. Tobacco leaf subepidermal cells introduced with *DlGLP5-8–2* recombinant plasmid, **G**-**J**. Tobacco leaf subepidermal cells introduced with *DlGLP1-5–1* recombinant plasmid, K-N. Tobacco leaf subepidermal cells introduced with pCAMBIA1302-35S-GFP empty vector epidermal cells; A, D, G, H, K, L are fluorescence excitation maps(A, D, G, K excitation wavelength HL: 488 nm; H, L excitation wavelength HL: 640 nm); B, E, I, M are bright field maps; C, F, G, N are superimposed maps

Based on the results of transcriptome data analysis and qRT‒PCR validation in longan somatic embryos, we selected two DlGLP genes, DlGLP5-8–2 and DlGLP1-5–1, with high expression levels but inconsistent expression trends, for subcellular localization. The prediction results showed that *DlGLP5-8–2* may localize in the cytoplasm and *DlGLP1-5–1* may localize in the extracellular stroma and chloroplasts. Using the GFP fusion protein expression method, the green fluorescence signal in tobacco leaf subepidermal cells was observed by fluorescence confocal microscopy. The results showed (Fig. 9) that the fluorescence in tobacco leaf subepidermal cells transfected with pCAMBIA1302-GFP was distributed throughout the cells, and *DlGLP5-8–2* localized in the cytoplasm, while *DlGLP1-5–1* localized in the extracellular stroma and chloroplasts, consistent with the predicted results, suggesting that *DlGLP5-8–2* and *DlGLP1-5–1* may be involved in material transport and regulation of cell growth [59]. The *OsGLP2-1*-eGFP fusion protein resembles a short needle or round object and shows rapid movement in the cytosol [16]. Although *DlGLP5-8–2* was not found to move rapidly in tobacco leaves in this study, round dot-like objects were observed in the cells (Fig. 9). Therefore, we speculated that *DlGLP5-8–2* may share functional similarities with *OsGLP2-1* and may be a component of nutrient uptake and transport during somatic embryo development and plant growth.

### *DlGLP1-5–1*-OE can increase the lignin content

**Fig. 10:**
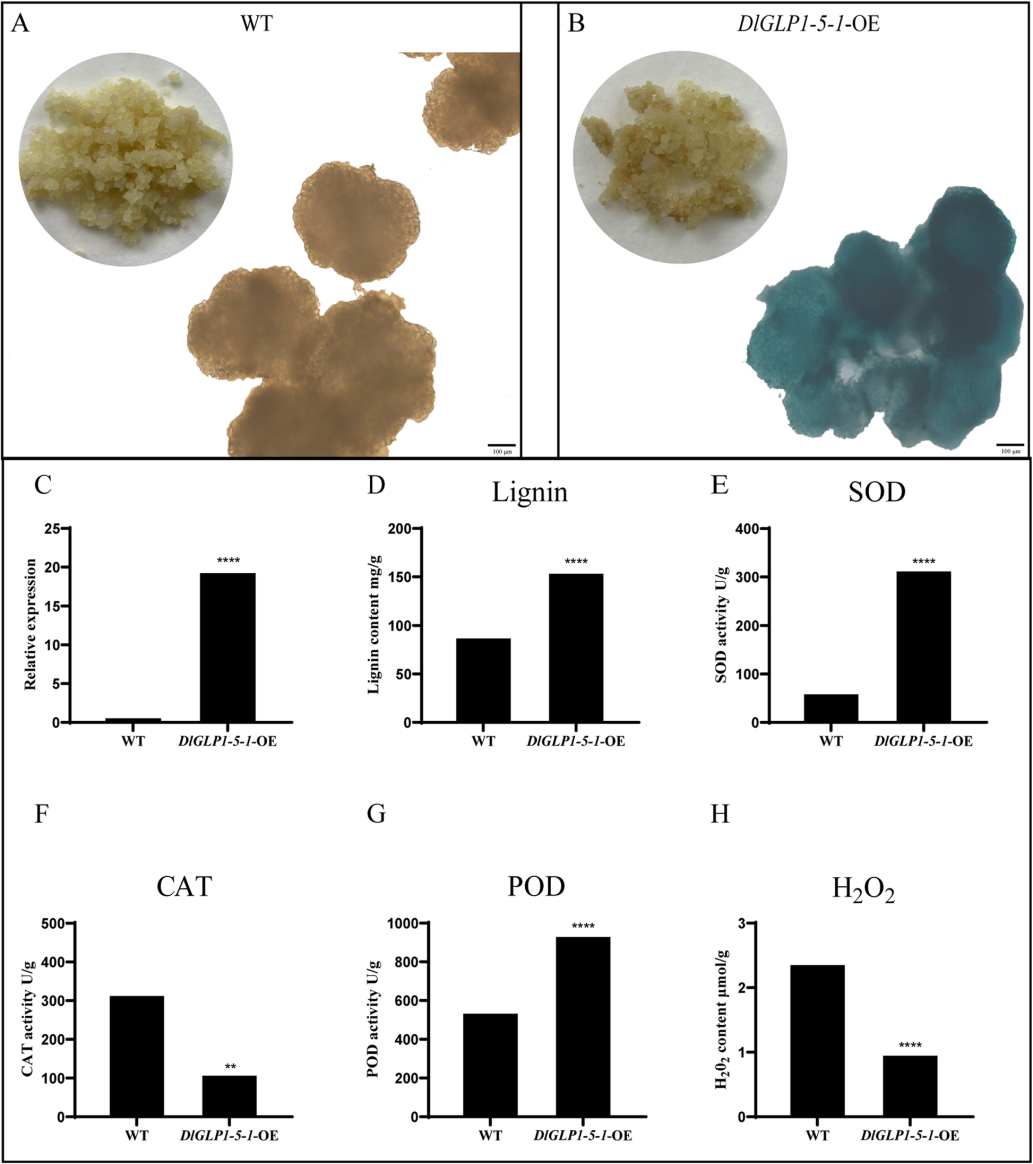
qPCR verification and physiological index determination of transgenic *DlGLP1-5–1*-OE. **A** GUS staining of Longan *DlGLP1-5–1* transgenic cell line, **B**. WT, **C**. qPCR verification of Longan *DlGLP1-5–1* transgenic cell line, **D**. Lignin content of Longan *DlGLP1-5–1* transgenic cell Line, **E**–**G**. Enzyme activity of Longan *DlGLP1-5–1* transgenic cell line, H. H_2_O_2_ content of Longan *DlGLP1-5–1* transgenic cell line

Longan GEs of *DlGLP1-5–1*-OE were obtained by *A. tumefaciens* transformation. Wild-type (WT) material was obtained from longan calli cultured in MS medium for 12 days. The longan GEs of *DlGLP1-5–1*-OE were yellow compared with those of WT, which may be related to the polyphenol oxidase activity of *DlGLP1-5–1*. Cell lines overexpressing *DlGLP1-5–1* were identified by GUS staining, and cell lines in the same state were selected for qRT‒PCR. GUS staining results showed that the cell line overexpressing *DlGLP1-5–1* was pure, and its gene expression level was 19 times higher than that of the WT (Fig. 10A).

Cell lines with high expression and good growth status were selected to further verify the function of *DlGLP1-5–1* in somatic embryos. According to previous studies, the GLP gene can participate in the formation of the cell wall and the synthesis of lignin by understanding the active oxygen species in plants [58]. Therefore, we measured the lignin content in the *DlGLP1-5–1*-OE cell line and found that the lignin content in the *DlGLP1-5–1*-OE cell line was significantly higher than that in the WT cell line (Fig. 10).

To further verify whether *DlGLP1-5–1* affects the lignin content by regulating ROS levels, we measured the activities of SOD, CAT and POD and the H_2_O_2_ content in the *DlGLP1-5–1*-OE cell line. The results showed that the activities of SOD and POD in the *DlGLP1-5–1*-OE cell line were significantly increased, but the activity of CAT and the H_2_O_2_ content were decreased. This indicates that *DlGLP1-5–1* may participate in lignin synthesis by regulating ROS levels (Fig. 10).

**Fig. 11:**
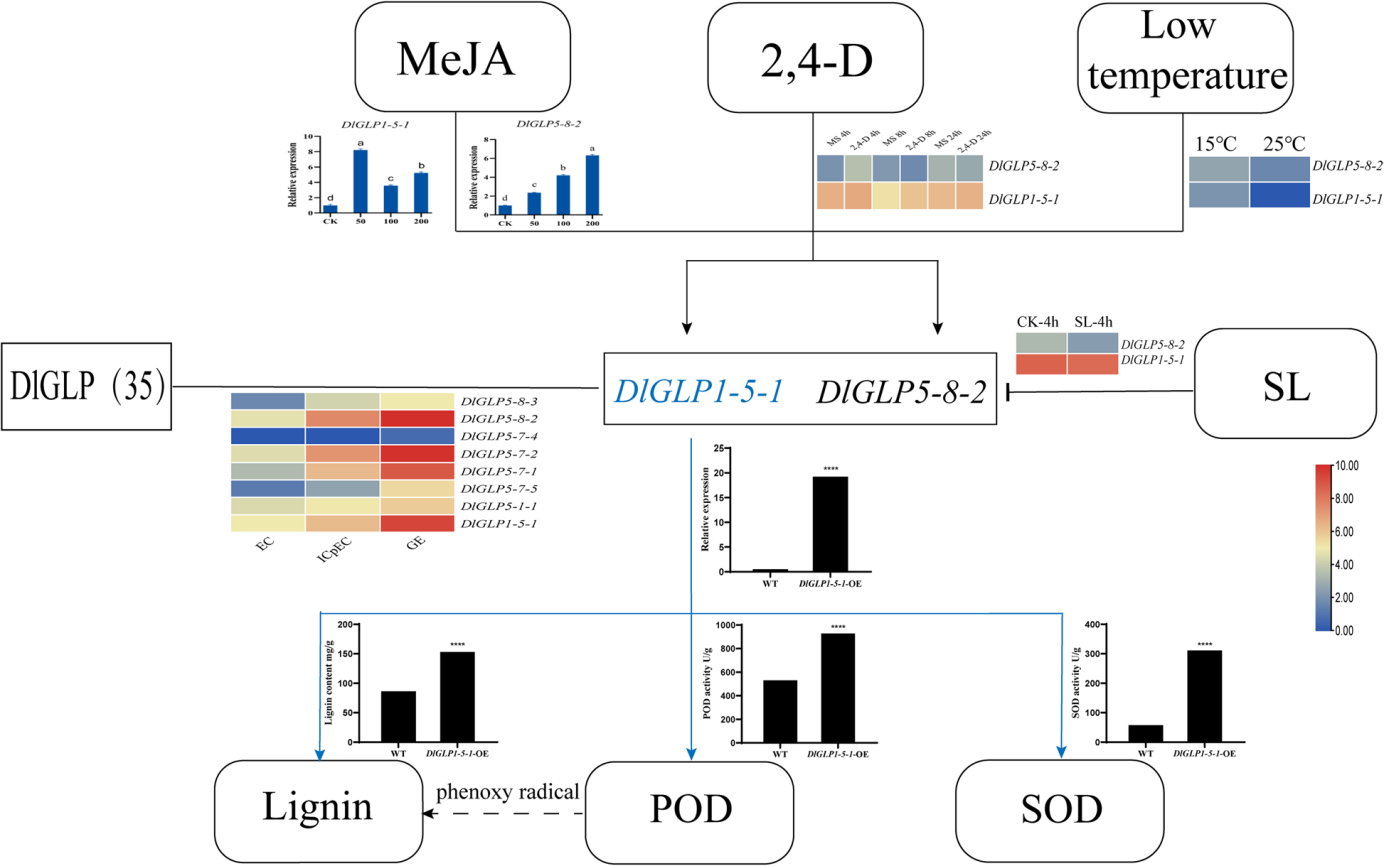
DlGLP gene expression and regulation pathway of overexpression *DlGLP1-5–1. *The arrow indicates promoting the process, and the “T” line indicates inhibiting the process

In conclusion, we drew a summary diagram based on the expression of the DlGLP gene in somatic embryos, the regulatory pathway of *DlGLP1-5–1*/*DlGLP5-8–2*, and the enzyme activity and lignin content under *DlGLP1-5–1* overexpression (Fig. 11).

## Discussion

### Structural and evolutionary analysis of the DlGLP genes and their role in the growth and development of longan

Germin-like proteins are involved in plant growth and developmental processes and respond to abiotic and biotic stresses [5, 22]. In total, 32, 44, 20 and 14 GLP family members have been identified in Arabidopsis [5], banana [6], cereals [6], and oilseed rape [11], respectively. In this study, genome-wide identification of the DlGLP gene family was performed, and 35 DlGLP family members were identified. Both *DlGLP5-8–2* and *DlGLP1-5–1* contain signal peptides, suggesting that *DlGLP5-8–2* may be involved in the synthesis and transport of glycans and lipids inside and outside the cell [59]. Transcriptomic analysis of different tissues of longan showed that DlGLPs were expressed in all tissues, and most DlGLPs were highly expressed in roots and seeds. The expression of lemon *ClGLP1* is increased during the fruit ripening process [60]. *DlGLP3-9–1* was highly expressed in young longan fruit and may play an important role in the longan fruit ripening process. The results suggest that DlGLPs may be involved in growth and development in longan, providing a theoretical basis for functional research on GLP genes in longan. It was recognized that tandem duplication events have mainly extended the GLP family members in D. longan. It seems that modifications in gene structure have affected the expression level and function of duplicated members [61, 62].

According to their evolutionary relationships, the GLP family members of longan, rice, Arabidopsis, lychee, banana and grape are divided into eight families. GLP genes may evolve into specific GLP genes in different species; some GLP genes may exist only in monocotyledons. Combined analysis of the gene structures revealed a high degree of similarity among the aggregated GLP genes, which may play roughly the same role in plants. Overexpression of *OsRGLP1* in tobacco significantly increased the H_2_O_2_ content in the leaves of tobacco transgenic plants [63]. The accumulation of H_2_O_2_ enhancements intercellular cross-linking, thus improving disease resistance in rice [23]. *AtGLP13* affects the cotyledon greening rate and main root growth in Arabidopsis [64]. The expression level of *GLP* genes in the fruit stalk tissue of 'SJM' longan was significantly and negatively correlated with the enzymatic activity of OXO [27]. *OsGLP2-1* is involved in the regulation of seed dormancy in rice [16]. GLP genes play an important role in plant growth and developmental processes, and studies on the growth and developmental stages of somatic embryos are still relatively scarce.

### DlGLPs may play an important role during the early stage of SE in longan

Germin-like proteins have been known to play roles during SE in plants. Repression of the *LmGER1* gene in hybrid larch led to a significant decrease in the efficiency of callus maturation and failure to form young plants [18]. However, knockdown of the *OsGLP1* gene in rice calli did not show the same effect as in hybrid larch [18]. In this experiment, we analyzed the expression of the DlGLP family during early SE in longan and found that some DlGLP genes were highly expressed in the ICpEC and GE stages. GLPs are involved in plant growth and development as SOD and OXO enzymes and generate H_2_O_2_, which can be involved in Ca^2+^ regulation and thus act on structural proteins of the cell wall [58]. These results demonstrate that DlGLPs may influence the differentiation of GEs in longan. However, the specific mechanism underlying the role of DlGLP genes during early SE in longan still needs further study.

### DlGLPs respond to the hormones MeJA, ABA and SL during early SE in longan

Analysis of promoter *cis-*acting elements showed that the GLP gene family members contain MeJA and ABA hormone-responsive elements, suggesting that the function of DlGLP genes may be related to hormone transduction in plants. Previous studies have found that *OsGLP2-1* is involved in the regulation of the ABA signaling pathway during rice seed primary dormancy, is directly regulated by ABI5 transcription factors and has been shown to be involved in the regulation of seed dormancy [16]. In Bowl Cress round (*Craterostigma*), *CpGLP1* localized to the cell wall and accumulated transiently under 100 μM ABA treatment [65]. ABA can promote somatic embryonic development in ginseng and spruce [66, 67]. Our results showed that the expression of DlGLPs was mostly inhibited under ABA hormone treatment. In particular, when the concentration was 100 μM, the expression of 7 DlGLPs decreased significantly. DlGLP gene expression was significantly upregulated under MeJA treatment. This indicates that DlGLP may participate in the growth and development process of longan at the SE stage by participating in the MeJA and ABA signaling pathways [31].

Our results also showed that some DlGLP genes responded to SL hormone regulation. Previous studies have shown that SL can reduce growth hormone transport by decreasing the accumulation of PIN proteins on the cytoplasmic membrane of thin-walled xylem tissues [68, 69]. It has been proven that the function of DlGLP genes is related to plant growth and development and cell wall formation, indicating that DlGLPs may participate in the signaling pathway of SL by regulating the changes in substances on the cytoplasmic membrane.

These results suggest that the signaling pathways of GLP genes involved in the response to hormones in plants are complex. This study provides a theoretical basis for further research on whether DlGLPs affect early SE in longan through their involvement in MeJA, ABA, and SL hormone transduction.

### *DlGLP1-5–1* promoted lignin accumulation

GLP genes can as act as SOD, OXO and serine protease inhibitors to participate in plant growth and development [14]. SOD activity was significantly increased after overexpression of *DlGLP1-5–1*, which was consistent with the result showing that GLP can act as SOD to participate in plant growth and development. These results indicate that *DlGLP1-5–1* plays an important role in ROS scavenging in the early stage of SE in longan. Previous studies have shown that POD can decompose H_2_O_2_ and produce phenoxy radicals to promote the synthesis of lignin [34]. After overexpression of *DlGLP1-5–1* in longan GEs, the POD activity and lignin content in longan globular embryos increased significantly. Therefore, it is speculated that overexpression of *DlGLP1-5–1* may promote lignin synthesis through phenoxy radicals produced by POD. The decrease in H_2_O_2_ content and CAT enzyme activity indicated that the lignin synthesis pathway involving *DlGLP1-5–1* is very complex. Previous studies have shown that the GLP gene has polyphenol oxidase activity, which can catalyze the synthesis of lignin [70]. Polyphenol oxidase is a plastid enzyme that may only be present in plastids [71]. The localization of *DlGLP1-5–1* in chloroplasts suggests that *DlGLP1-5–1* may have polyphenol oxidase activity. Whether *DlGLP1-5–1* acts as a catalyst for lignin synthesis by polyphenol oxidase remains to be further studied.

Overexpression of *DlGLP1-5–1* can significantly increase lignin accumulation in longan somatic embryos, which indicates that *DlGLP1-5–1* plays an important role in lignin synthesis in longan. Therefore, it is speculated that *DlGLP1-5–1* plays a similar role in other species, which provides a reference for further research on the biosynthetic pathway of lignin. At the same time, it also provides a reference for improving lignin accumulation through genetic engineering in the agricultural industry.

## Conclusions

In this study, 35 DlGLP genes were obtained and divided into eight different subfamilies in longan. Gene sequence analysis showed that the DlGLP gene was highly conserved during evolution. The exon–intron and motif compositions of different subfamilies were similar. By transcriptome analysis, we found that DlGLP genes may widely participate in various plant growth and developmental processes and play important roles during early SE and root formation in longan. Gene expression analysis showed that DlGLPs were regulated by ABA, MeJA and SL and may play roles during early SE in longan by responding to hormone signal pathways. In addition, *DlGLP5-8–2* and *DlGLP1-5–1* were located in the cytoplasm and extracellular matrix/chloroplast, respectively, indicating that they may participate in the regulation of material transport and cell growth. Overexpression of *DlGLP1-5–1* in the GE stage increases the lignin content and SOD and POD activities. However, the decrease in CAT activity and H_2_O_2_ content indicates that *DlGLP1-5–1* has a complex regulatory mechanism in lignin synthesis. This study provides a reference for future research related to the functional analysis of DlGLPs and for improving lignin accumulation through genetic engineering in the agricultural industry.

## Supplementary Information

**Addiional file 1: Table 1.** qRT- PCR primer sequence. **Table 2.** Gene cloning and subcellular localization primer sequences. **Table 3.** Expression of DlGLP in different tissues of ‘SJM’ Longan. **Table 4.** Expression of DlGLP genes in early somatic embryogenesis of longan. Expression of DlGLP genes in EC under 2,4-D treatment. **Table 5.** Expression of DlGLP genes in EC under temperature and SL treatment.

## Data Availability

All data used in the document have been uploaded to the NCBI database, such as the login number in the document is unavailable. You can view through the following reviewer links. The transcriptome data involved in Fig. 6 are also provided in S3, S4, S5 and S6 in the supplementary table. Longan genome of the third generation:https://dataview.ncbi.nlm.nih.gov/object/PRJNA792504?reviewer=icn3dhkgoj84q493bbkjivtnlq Temperature treated transcriptome:https://dataview.ncbi.nlm.nih.gov/object/PRJNA889670?reviewer=vji3gtv0bnskkduqjdrr1pg4rh Longan somatic embryo transcriptome of the third generation:https://dataview.ncbi.nlm.nih.gov/object/PRJNA891444?reviewer=qjiufii6hibgvo4vqcf1c110gb Longan 2, 4-D transcriptome:https://dataview.ncbi.nlm.nih.gov/object/PRJNA889205?reviewer=cal86jregmb80p2lb35qr72v2o Longan SL transcriptome:https://dataview.ncbi.nlm.nih.gov/object/PRJNA889220?reviewer=tp58rvbrrn64jpd8tboufa34mc All experiments and analyses in this paper are based on plants and do not involve moral and ethical issues. Therefore, no moral and ethical consent is required.
